# Interactions Between a Belowground Herbivore and Primary and Secondary Root Metabolites in Wild Cabbage

**DOI:** 10.1007/s10886-015-0605-7

**Published:** 2015-08-14

**Authors:** Moniek Van Geem, Jeffrey A. Harvey, Anne Marie Cortesero, Ciska E. Raaijmakers, Rieta Gols

**Affiliations:** Department of Terrestrial Ecology, Netherlands Institute of Ecology (NIOO-KNAW), Wageningen, The Netherlands; Department of Ecological Sciences, Section Animal Ecology, VU University, Amsterdam, The Netherlands; Institute of Genetics, Environment and Plant Protection, Rennes University, Rennes, France; Laboratory of Entomology, Wageningen University, Wageningen, The Netherlands

**Keywords:** Amino acid, Belowground, *Brassica oleracea*, *Delia radicum*, Induction, Root chemistry, Sugar

## Abstract

Plants are attacked by both above- and belowground herbivores. Toxic secondary compounds are part of the chemical defense arsenal of plants against a range of antagonists, and are subject to genetic variation. Plants also produce primary metabolites (amino acids, nutrients, sugars) that function as essential compounds for growth and survival. Wild cabbage populations growing on the Dorset coast of the UK exhibit genetically different chemical defense profiles, even though they are located within a few kilometers of each other. As in other Brassicaceae, the defensive chemicals in wild cabbages constitute, among others, secondary metabolites called glucosinolates. Here, we used five Dorset populations of wild cabbage to study the effect of belowground herbivory by the cabbage root fly on primary and secondary chemistry, and whether differences in chemistry affected the performance of the belowground herbivore. There were significant differences in total root concentrations and chemical profiles of glucosinolates, amino acids, and sugars among the five wild cabbage populations. Glucosinolate concentrations not only differed among the populations, but also were affected by root fly herbivory. Amino acid and sugar concentrations also differed among the populations, but were not affected by root fly herbivory. Overall, population-related differences in plant chemistry were more pronounced for the glucosinolates than for amino acids and sugars. The performance of the root herbivore did not differ among the populations tested. Survival of the root fly was low (<40 %), suggesting that other belowground factors may override potential differences in effects related to primary and secondary chemistry.

**Electronic supplementary material:**

The online version of this article (doi:10.1007/s10886-015-0605-7) contains supplementary material, which is available to authorized users.

## Introduction

The study of plant-insect interactions is a foundation for understanding evolutionary and community ecology (Schoonhoven et al. 2005). Early studies on plant-insect interactions focused primarily on the aboveground compartment, ignoring the fact that plants, through their roots, also interact with the biotic environment belowground. The importance of biotic interactions in the rhizosphere has become apparent in the past two decades (Masters and Brown 1992; Masters et al. 1993; van der Putten et al. 2001). Moreover, the belowground environment has consequences for biotic interactions with aboveground plant tissues and *vice versa* (Wardle et al. 2004). Plants are attacked by insect herbivores both in the aboveground and belowground domains, often simultaneously. It has been shown that belowground herbivores, by removing root tissues, negatively affect the functioning of roots, for instance through a reduction in the uptake and storage of nutrients, which cascade to other plant tissues thereby affecting the whole plant (Blossey and Hunt-Joshi 2003; van der Putten 2003).

Plants tissues produce both primary and secondary chemical compounds (metabolites) that have different biological functions. Primary metabolites are those that plants need in order to grow, develop, and reproduce, and include amino acids and sugars (Bidwell 1974; Gibson 2005). Plants also produce secondary metabolites that protect organs, especially those that are important for survival and reproduction, against herbivores and pathogens. Secondary metabolites also are used to complete with other plants, to attract pollinators and seed dispersers, to mitigate symbiotic interactions, and to protect against UV-light or other physical stress (Wink 1999). The balance between concentrations of secondary and primary metabolites is a determinant of food plant quality for insect herbivores (Awmack and Leather 2002; Scriber and Slansky 1981). Plant primary metabolites provide essential nutrients for insect development, whereas secondary metabolites can be repellent and/or toxic for many insects and thus interfere with insect behavior and physiology (Schoonhoven et al. 2005; Scriber and Slansky 1981). However, for many co-evolved specialist herbivores, host-derived secondary metabolites function as feeding or oviposition stimulants (Schoonhoven et al. 2005).

Plant secondary chemistry is phylogenetically conserved, and genetic variation among populations often is maintained over different scales of space and time (Agrawal et al. 2012; Berenbaum and Zangerl 1991; Hartmann 1996; Hoy et al. 1998; van Geem et al. 2013). Various studies have shown that concentrations and specific compounds differ among species within a plant family, individuals and populations within a species, and even plant structures of individual plants (Fahey et al. 2001; Fordyce and Malcolm 2000; Gols et al. 2008b; Häring et al. 2007; Hartmann 1996). Thus far, most studies on the evolution of diversity in secondary metabolites driven by insect herbivores have focused on aboveground plant tissues. Less is known about variation in defense chemistry in the rhizosphere and effects of defensive chemistry on belowground herbivores. Moreover, intra-specific variation in primary chemistry has been virtually ignored.

Wild cabbage (*Brassica oleracea*) plants grow naturally along the Atlantic coasts of north-western Europe and belong to the large family Brassicaceae. Plant species within this family all produce glucosinolates (hereafter GS), inducible secondary metabolites that play a role in mediating plant-insect interactions (Gols et al. 2009; Hopkins et al. 2009). GS profiles not only differ among populations (Gols et al. 2008b; Mithen et al. 1995; Moyes et al. 2000; Newton et al. 2009; van Geem et al. 2013), but also between individual plants within a population (Mithen et al. 1995) and between different plant organs of individual plants (Bennett and Wallsgrove 1994). Variation in defense chemistry profiles makes the English wild cabbage populations a good model system for studying the effect of plant secondary metabolites on insect performance while incorporating genetic variation that is maintained over a limited spatial scale (*e.g.* 20 km).

Induction of GS has been well-studied in aboveground plant tissues (Agrawal et al. 1999; Agrawal 2000; Gols et al. 2008a, b; Harvey et al. 2007, 2011; Poelman et al. 2008) but less so in belowground tissues (Soler et al. 2005; van Dam and Raaijmakers 2006). Moreover, the number of studies investigating the combined effect of primary and secondary metabolites in leaves on insect performance in wild plants are limited (but see Cole 1997; Wurst et al. 2006). Even less is known about variation in primary and secondary chemical profiles in roots, their effect on root insect development, and whether concentrations of these chemicals change in response to belowground herbivory, which is the topic of this study.

The main aim of our study was to determine whether the chemical profiles of primary and secondary metabolites in roots differed among the wild cabbage populations in response to belowground herbivory. We also were interested whether there was a link between root fly performance and root chemistry. In a greenhouse experiment, we grew plants from seeds collected from five naturally growing populations in Dorset, England that differ in foliar GS chemistry (Gols et al. 2008b; Newton et al. 2010). We compared development of a specialist herbivore, the cabbage root fly *Delia radicum* L. (Diptera: Anthomyiidae) on these plants. In addition to root chemistry, we also measured root biomass and insect performance variables. Given that previous studies have shown that there is constitutive and inducible variation in GS chemistry in leaf tissues of the cabbage populations, we hypothesize that: 1) this variation also is present in the roots, and that 2) the performance of root flies would differ when grown on different populations, as has been demonstrated with aboveground insects.

## Methods and Materials

### Plants

**Fig. 1:**
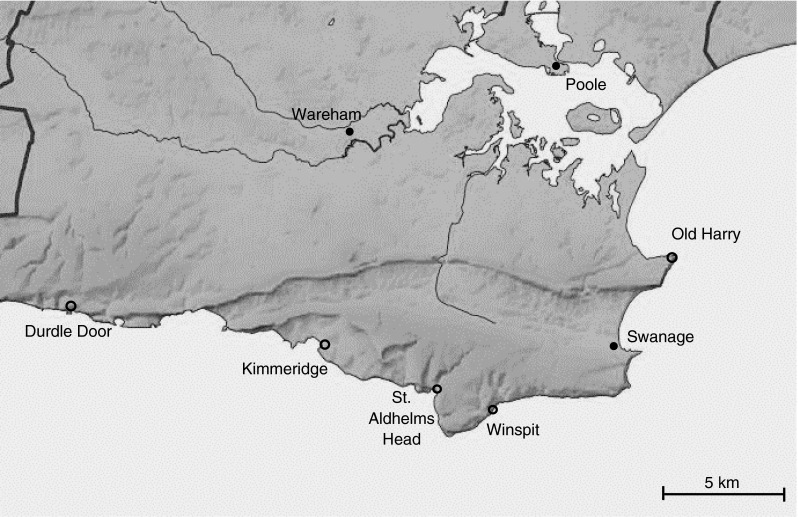
Locations where the five wild cabbage (*Brassica oleracea*) populations (*open dots*) are located in Dorset, UK

Wild cabbage (*Brassica oleracea*) seeds were collected from five populations that are located on the southern coast of the UK, in the Dorset area near Swanage (Fig. 1). Seeds were collected from multiple plant individuals per population. The five populations are Durdle Door (DD; 50°62′N, 2°27′W), Kimmeridge (KIM; 50°35′N, 2°03′W), St. Aldhelms Head (SAH; 50°69′N, 2°05′W), Winspit (WIN; 50°34′N, 2°02′W), and Old Harry (OH; 50°38′N, 1°55′W). Each population has a unique microhabitat; WIN is sheltered from the prevailing southwest wind, OH and DD are partially wind-exposed, whereas KIM and SAH plants are exposed to the wind because they grow on top of the cliffs facing south to southwest. Differences in microhabitat affect the herbivore pressure at each location, since highly exposed locations are likely to be less accessible to herbivores than secluded ones.

Seeds were germinated in germination soil (Lentse Stekgrond, Lent, The Netherlands), and after one week seedlings were transplanted into individual 2-L pots containing a soil mixture of 11 % clay, 69 % peat, and 20 % pumice. Plants were grown in a glasshouse at 21 ± 1 °C (day) and 16 ± 1 °C (night) with 70 % r.h. and a L16:D8 light regime. They were watered every other day and given extra nutrients twice a week using half-strength Hoagland solution (Hoagland and Arnon 1950). Plants were 8 wk old when they were used in the experiments.

### Insects

The cabbage root fly (*Delia radicum*) is a common pest species in agriculture and a specialist on plant species from the Brassicaceae family. Female flies lay their eggs near the stem-root interface, and newly hatched larvae crawl into the soil and burrow into the upper roots. Larvae feed on root tissue and can have severe negative effects on growth of their host plant.

The root fly culture was established at the NIOO in Wageningen from root fly pupae that were provided by the University of Rennes, France. That colony was started in September 2009 with flies collected in the field (Le Rheu, Brittany, France, 48°07016″N, 01°47041″W). The root flies were reared in a climate room (21 ± 1 °C, 50–70 % r.h., L16:D8) on a diet of sugar, milk powder, and nutritional yeast (1:1:1) and maintained on swedes or turnips. Water was provided through humidified cotton balls. To obtain larvae, adult root flies were offered a fresh piece of turnip placed in a 9 cm Petri dish with moist filter paper as an oviposit site. After 1 day, eggs were collected and kept on moist filter paper until they developed into first instar larvae, which were used to inoculate plants.

### Experimental Design

From each population, 20 plants were assigned to the root fly induction (= belowground herbivory) treatment and 10 plants to the control (= no herbivory) treatment. Newly hatched root fly larvae, 3 per plant, were transferred carefully to the stem/root interface of plants with a small brush. After transfer, larvae were observed to confirm that they crawled into the soil. Two weeks later, every plant was put into an individual sleeve cage (48 × 71 cm, 104 × 94 mesh; Bugdorm, MegaView Science, Taiwan) to contain emerging adult root flies.

Emergence of adults was recorded three times a day, and their development time was determined as the number of days between egg hatching and adult eclosion. Survival was calculated as the number of flies per plant that emerged from the three fly larvae that were initially introduced. Adults were killed immediately after eclosion by freezing at −20 °C. Their dry body mass was obtained by drying them in an oven at 70 °C for 3 days. After all flies emerged, plants were harvested to record root biomass (clean roots were weighed prior to storage), and the roots of plants were sampled to analyze glucosinolates, amino acids, and sugars (described below).

### Chemical Root Analysis

Roots of all 30 plants per population, 150 in total, were removed from the soil, washed, placed in paper bags, and stored at −20 °C until further processing. Roots were freeze-dried for 4 days. Dried roots then were cut up into small pieces, and a representative sample (parts from both thick and thin roots) was selected for further analysis. These samples were ground to powder with a grinding machine (Retch, type MM 301).

For the methanol extraction of GS, sugars, and amino acids, approximately 50 mg of the ground root were weighed in a 2 ml microfuge tube. Samples were suspended in 1 ml 70 % MeOH, and vortexed before boiling in a water bath for 5 min. Samples were transferred to an ultrasonic bath for 15 min and centrifuged for 10 min at 10,000 rpm to obtain clear supernatant solutions that were transferred to clean microfuge tubes. The extraction was repeated, and the second extract for each sample was pooled with the first.

The mass of each extract was adjusted to be equal (+/−0.01 g) to the average mass of a standard 2 ml aliquot of 70 % MeOH.

### Glucosinolate Analysis

One ml of the extract was added to a DEAE-Sephadex A25 column, followed by two additions of 1 ml of 70 % MeOH, one addition of 1 ml of MilliQ water, and two additions of 1 ml of 20 mM sodium acetate buffer (pH = 5.5). Then 20 μl (eight units) of sulfatase (Sigma type H-1 from Helix pomata) in aqueous solution was added to each column followed by 50 μl of 20 mM sodium acetate buffer. The columns were placed over vials, covered with aluminum foil, and left to stand overnight. The next day, the desulfoglucosinolates were eluted from each column by washing with 1 ml of MilliQ water twice, and the samples were freeze dried. Each freeze dried sample was redissolved in 500 μl MilliQ water and pressed through a 0.2 μm nylon syringe filter into an HPLC vial.

High-performance liquid chromatography (HPLC) was used to measure concentrations of GS, sugars, and amino acids. The GS analyses were performed with an Alltima C18 column (150 × 4.6 mm) using 50 μl injection volume. The column was kept at a temperature of 40 °C, and the flow rate was 0.75 ml/min. Glucosinolates were detected with a UV diode array at a wavelength of 229 nm. Sinigrin in five different concentrations (63–625 μM) was used as an external standard for the quantification of GS. Individual GS were identified based on their retention times and UV spectra compared to those of the standards (EC Community Bureau of Reference, Brussels, Belgium, BCR-367R). Final concentration (nMoles/mg) were calculated by correcting for the volume and dry mass of the extract and original tissue.

### Soluble Sugar Analysis

Ten μl of the stock extract was mixed with 990 μl MilliQ water in an HPLC vial. Samples were analyzed on the ion-exchange HPLC with a CarboPac PA1 main column (2 × 250 mm) and a CarboPac PA1 guard column (2 × 50 mm) using 5 μl injection volume. The columns were kept at a temperature of 20 °C, and the flow rate was 0.25 ml/min. The standard curves of 11 sugars (2.5–10 ppm) were used as reference. Final concentrations (μg/mg) of sugars in the roots were calculated by correcting for the volume and dry mass of the extract and the original tissue.

### Amino Acid Analysis

Twenty μl of the stock extract were mixed with 980 μl MilliQ water in an HPLC vial. Samples were analyzed on the ion-exchange HPLC with an AminoPac PA10 main column (2 × 250 mm) and an AminoPac PA10 guard column (2 × 50 mm) using 25 μl injection volume. The column was kept at a temperature of 30 °C, and the flow rate was 0.25 ml/min. The standard curves of 20 amino acids (1–8 μM) were used as reference. Final concentrations (nMoles/mg) of amino acids in the roots were calculated by correcting for the volume and dry mass of the extract and the original tissue.

### Statistical Analysis

All univariate analyses were performed using SPSS version 22 (IBM SPSS Statistics) and the multivariate analyses with Canoco version 5.03 (ter Braak and Šmilauer, The Netherlands).

Total metabolite levels and the root biomass data were analyzed using two-way ANOVA with plant population and root fly treatment, as well as their interaction, as fixed factors. When needed, data were log- or square-root-transformed to meet assumptions of normality and homogeneity of variance. *Post-hoc* Tukey multiple comparison tests were performed when the ANOVA models were significant.

Chemical profiles of the roots of all plant populations were analyzed using multivariate principal component analysis (PCA) and redundancy analysis (RDA) to test for differences between plant populations and treatments. RDA is a linear method of canonical ordination also described as a direct gradient analysis technique that summarizes linear relationships between response variables (here, concentrations of chemicals) that are explained by a set of variables (here, populations and treatments) (Lepš and Šmilauer 2003).

Concentrations of chemicals were log-transformed and mean-centered. Correlations between the chemical data and the insect performance and root biomass data were tested with RDA in Canoco. Adult body mass and development time were tested separately for female and male root flies.

The root fly performance parameters development time and adult body mass were analyzed using a linear mixed model with plant population and sex as fixed factors. Plant ID was used as a random factor to deal with the fact that the three data points (three root flies) per plant were not independent. Insect survival data were analyzed with binary logistic regression.

## Results

### Glucosinolates

Thirteen different GS were present in root tissues of all five plant populations (Table 1). Based on their amino acid precursors, GS can be classified into three classes: aliphatic, indole, and aromatic (Halkier and Gershenzon 2006). Eight of the GS belonged to the aliphatic, four to indole, and one to aromatic GS class (see Table 1).

**Table 1:** Mean concentrations (±SE) of individual glucosinolates (GS) (μmol/g), sugars (mg/g) and amino acids (μmol/g) of control plants and induced plants for the five wild cabbage populations

Compound	Wild cabbage population									
	DD		KIM		SAH		WIN		OH	
	Control	Induced	Control	Induced	Control	Induced	Control	Induced	Control	Induced
Glucosinolates										
Aliphatic GS										
Glucoalyssin	0.01 ± 0.01	0.03 ± 0.01	–	0.01 ± 0.00	0.07 ± 0.01	0.01 ± 0.01	0.12 ± 0.03	0.08 ± 0.02	0.06 ± 0.01	0.05 ± 0.01
Glucobrassicanapin	0.11 ± 0.05	0.08 ± 0.05	–	0.01 ± 0.00	0.06 ± 0.04	0.09 ± 0.02	0.14 ± 0.06	0.11 ± 0.02	–	0.02 ± 0.01
Glucoerucin	4.91 ± 0.72	4.36 ± 0.65	1.74 ± 0.35	1.82 ± 0.39	8.60 ± 1.02	5.10 ± 0.58	4.14 ± 1.45	7.22 ± 1.25	7.22 ± 1.62	5.70 ± 0.90
Glucoiberin	0.05 ± 0.05	0.33 ± 0.11	0.82 ± 0.09	0.94 ± 0.09	0.25 ± 0.10	0.35 ± 0.08	0.15 ± 0.15	0.26 ± 0.08	0.53 ± 0.17	0.40 ± 0.09
Gluconapin	2.37 ± 0.40	3.66 ± 0.57	0.75 ± 0.22	1.55 ± 0.24	36.96 ± 6.29	39.25 ± 3.81	42.69 ± 7.53	39.35 ± 3.71	10.40 ± 3.14	8.95 ± 1.72
Glucoraphanin	4.23 ± 0.34	3.72 ± 0.31	1.72 ± 0.20	1.57 ± 0.17	4.29 ± 0.31	2.97 ± 0.28	3.31 ± 0.61	3.18 ± 0.30	4.02 ± 0.32	2.95 ± 0.29
Progoitrin	62.94 ± 5.71	46.01 ± 5.60	7.56 ± 1.72	8.96 ± 1.45	10.19 ± 2.20	9.92 ± 1.30	10.84 ± 1.33	8.40 ± 0.66	10.70 ± 2.59	9.48 ± 1.63
Sinigrin	0.95 ± 0.86	4.54 ± 1.33	6.60 ± 1.00	9.27 ± 0.81	3.63 ± 1.46	8.08 ± 2.24	1.08 ± 0.85	5.00 ± 1.24	5.12 ± 1.41	5.01 ± 0.98
Indole GS										
4-Methoxyglucobrassicin	1.84 ± 0.40	0.84 ± 0.14	0.82 ± 0.15	0.90 ± 0.14	0.82 ± 0.28	1.22 ± 0.16	1.65 ± 0.26	1.38 ± 0.18	1.55 ± 0.29	1.24 ± 0.17
4-Hydroxyglucobrassicin	1.36 ± 0.21	1.14 ± 0.23	0.42 ± 0.07	2.06 ± 0.35	0.32 ± 0.09	0.36 ± 0.05	0.11 ± 0.02	0.22 ± 0.04	0.46 ± 0.10	0.44 ± 0.09
Glucobrassicin	0.98 ± 0.18	0.49 ± 0.07	0.54 ± 0.09	0.59 ± 0.12	0.45 ± 0.14	0.67 ± 0.09	1.09 ± 0.31	0.69 ± 0.08	1.17 ± 0.23	0.75 ± 0.13
Neoglucobrassicin	10.11 ± 2.60	6.29 ± 1.16	4.35 ± 0.69	5.36 ± 1.40	2.12 ± 0.44	4.25 ± 0.88	12.02 ± 2.57	8.34 ± 1.05	3.72 ± 0.83	3.42 ± 0.66
Aromatic GS										
Gluconasturtiin	19.83 ± 1.64	18.27 ± 1.43	7.81 ± 0.81	11.79 ± 0.83	13.73 ± 1.35	11.07 ± 1.04	11.21 ± 1.83	12.43 ± 1.11	9.60 ± 1.16	8.00 ± 0.76
Sugars										
Glucose	1.48 ± 0.15	1.32 ± 0.09	1.40 ± 0.22	1.07 ± 0.10	1.59 ± 0.22	1.34 ± 0.13	1.39 ± 0.17	1.24 ± 0.11	1.28 ± 0.17	1.44 ± 0.11
Fructose	0.46 ± 0.09	0.54 ± 0.06	0.89 ± 0.17	0.60 ± 0.10	1.10 ± 0.25	0.89 ± 0.10	0.91 ± 0.16	0.77 ± 0.18	0.73 ± 0.08	0.98 ± 0.10
Manitol	–	0.01 ± 0.01	0.02 ± 0.02	0.003 ± 0.003	0.003 ± 0.003	0.01 ± 0.00	0.02 ± 0.01	0.01 ± 0.01	0.02 ± 0.02	0.03 ± 0.01
Sorbitol	0.56 ± 0.01	0.60 ± 0.01	0.81 ± 0.08	0.58 ± 0.01	0.76 ± 0.03	0.73 ± 0.02	0.66 ± 0.04	0.69 ± 0.03	0.76 ± 0.01	0.88 ± 0.04
Sucrose	20.44 ± 1.62	20.11 ± 1.49	25.34 ± 2.49	21.36 ± 1.67	30.44 ± 2.74	24.82 ± 1.99	16.27 ± 1.62	18.50 ± 0.79	19.94 ± 3.03	25.21 ± 1.74
Trehalose	0.04 ± 0.02	0.06 ± 0.04	0.02 ± 0.02	0.02 ± 0.01	–	0.07 ± 0.02	0.14 ± 0.05	0.06 ± 0.02	0.09 ± 0.04	0.07 ± 0.02
Amino acids										
Arginine	38.45 ± 2.70	38.56 ± 2.70	46.64 ± 4.19	36.87 ± 2.30	39.21 ± 1.74	39.35 ± 1.88	29.52 ± 4.15	30.72 ± 1.73	43.44 ± 4.11	48.76 ± 2.77
Aspargine	1.58 ± 0.12	1.62 ± 0.21	2.65 ± 0.12	2.25 ± 0.25	1.86 ± 0.15	2.41 ± 0.23	2.28 ± 0.32	2.13 ± 0.11	1.89 ± 0.18	1.94 ± 0.24
Glutamine	2.66 ± 0.30	2.54 ± 0.34	4.14 ± 0.52	3.50 ± 0.39	4.02 ± 0.67	4.49 ± 0.45	4.47 ± 0.82	3.67 ± 0.38	3.35 ± 0.61	3.94 ± 0.84
Histidine	3.36 ± 0.56	4.04 ± 0.66	6.43 ± 0.64	6.41 ± 0.45	3.47 ± 0.83	6.28 ± 1.08	4.87 ± 1.01	5.56 ± 0.54	6.00 ± 0.86	4.84 ± 0.68
Isoleucine	300.7 ± 17.7	296.5 ± 17.6	414.2 ± 21.4	335.3 ± 24.2	450.1 ± 29.0	415.7 ± 25.8	282.9 ± 30.6	284.2 ± 12.3	315.9 ± 39.3	377.9 ± 21.9
Proline	0.60 ± 0.18	1.11 ± 0.25	1.70 ± 0.34	1.84 ± 0.34	0.38 ± 0.19	0.93 ± 0.29	0.26 ± 0.14	0.64 ± 0.26	0.29 ± 0.17	0.44 ± 0.15
Serine	2.13 ± 0.29	2.35 ± 0.27	4.10 ± 0.35	3.95 ± 0.28	2.72 ± 0.46	3.33 ± 0.48	1.63 ± 0.27	2.40 ± 0.29	2.47 ± 0.33	2.58 ± 0.30
Threonine	10.62 ± 0.97	10.08 ± 0.70	11.46 ± 1.67	8.49 ± 0.77	11.48 ± 1.62	10.81 ± 0.95	11.18 ± 1.34	9.23 ± 0.78	10.14 ± 1.32	10.79 ± 0.79
Tyrosine	0.66 ± 0.04	0.67 ± 0.04	0.54 ± 0.07	0.56 ± 0.03	0.52 ± 0.07	0.57 ± 0.04	0.39 ± 0.05	0.38 ± 0.03	0.56 ± 0.03	0.55 ± 0.04

**Fig. 2:**
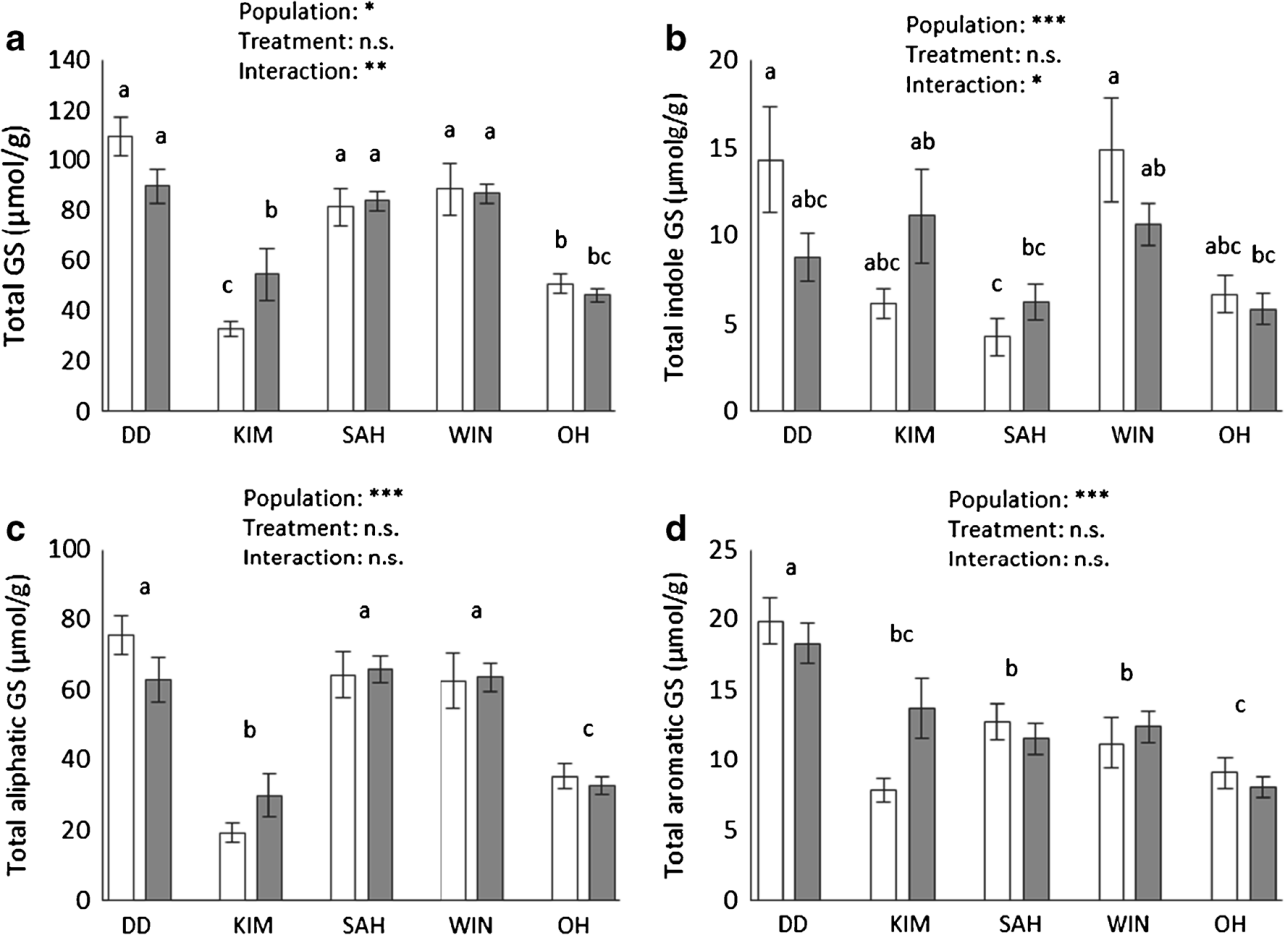
Concentrations (mean ± SE) of total glucosinolates (GS) (**a**), indole GS (**b**), aliphatic GS (c), and aromatic GS (D) in control plants (*white bars*) and in plants that were damaged by cabbage root fly larval feeding (*grey bars*) in root tissues of the five wild cabbage populations. *** *P* ≤ 0.001, ** *P* ≤ 0.01, * *P* ≤ 0.05, *n.s.* non-significant

The effect of belowground herbivory on total GS concentration in the roots was dependent on the plant population tested (plant population: *F*_4,136_ = 45.1, *P* < 0.001; treatment: *F*_1,136_ = 0.261, *P* = 0.610; interaction: *F*_4,136_ = 3.59, *P* = 0.008). Belowground herbivory increased total GS concentration in KIM roots, but not in roots of the other populations (Fig. 2a). Total GS concentrations tended to be higher in DD, SAH, and WIN than in KIM and OH plants.

Analysis of total concentrations of the three GS classes separately showed an interaction effect between population and root fly treatment for the indole GS (*F*_4,136_ = 2.57, *P* = 0.041; Fig. 2b), and a population effect for the aliphatic GS (*F*_4,136_ = 50.093, *P* ≤ 0.001; Fig. 2c) and the aromatic GS (*F*_4,135_ = 16.727, *P* ≤ 0.001; Fig. 2d). Although the effect of belowground herbivory on the indole GS concentrations in the roots significantly varied between the populations, no plant population showed a significant increase or reduction of indole GS. Similar to concentrations of total GS, the concentrations of aliphatic GS were highest in DD, SAH, and WIN, and lower in KIM and OH. The only aromatic GS, gluconasturtiin, had the highest concentrations in DD and lowest in OH.

**Fig. 3:**
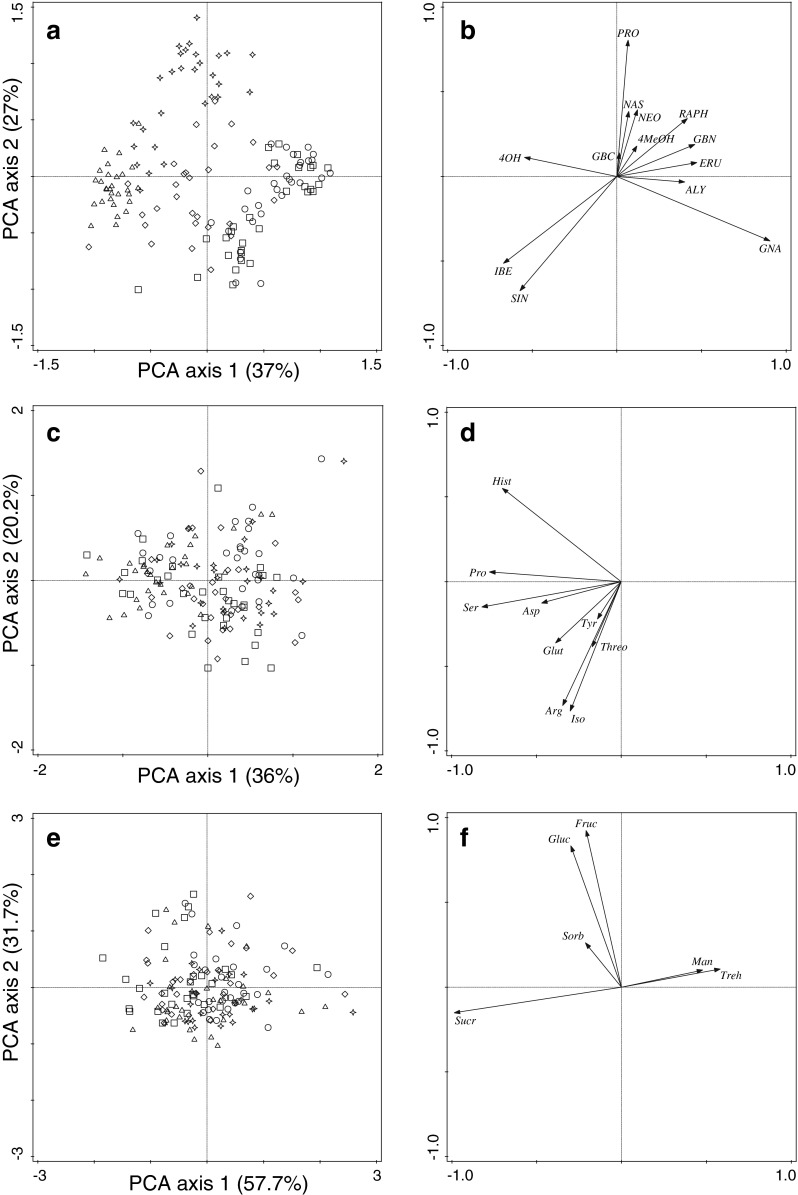
PCA ordination plots and loading plots of the glucosinolate profiles (**a** and **b**), amino acid profiles (**c** and **d**), and sugar profiles (**e** and **f**) of the five wild cabbage populations (control and root fly damaged samples both included). The percentage explained variation is given for each axis between parentheses. Populations: DD: star, KIM: triangle, SAH: square, WIN: circle, OH: diamond. *Abbreviations*: Aliphatic Glucosinolates (GS): ALY = glucoalyssin, EUR = glucoerucin, GBN = glucobrassicanapin, GNA = gluconapin, PRO = progoitrin, RAPH = glucoraphanin, SIN = sinigrin; Indole GS: 4MeOH = 4-methoxyglucobrassicin; 4OH = 4-hydroxyglucobrassicin, GBC = glucobrassicin, NEO = neoglucobrassicin; Aromatic GS: NAS = gluconasturtiin; Amino acids: Arg = arginine, Asp = asparagine, Glut = glutamine, Hist = histidine, Iso = isoleucine, Pro = proline, Ser = serine, Threo = threonine, Tyr = tyrosine; Sugars: Fruc = fructose, Gluc = glucose, Man = mannitol, Sorb = sorbitol, Sucr = sucrose, Treh = trehalose

PCA of individual GS concentrations in roots showed that there was some degree of separation between the GS profiles of the five plant populations (Fig. 3a and b). Four sample clusters are distinguishable from the figure: KIM, DD, OH, and SAH together with WIN. The GS profile of DD was characterized by high concentrations of progoitrin (60 % of the total GS concentration), whereas in SAH and WIN, gluconapin was the most prominent GS, constituting almost 50 % of the total GS concentration. Root tissues of KIM and OH plants did not have a dominant GS, but contained several at moderately high concentrations (progoitrin, sinigrin, and gluconasturtiin in KIM, and progoitrin, gluconapin, and gluconasturtiin in OH). Except for gluconasturtiin, which contributed considerably to GS content (KIM: 24–25 %, OH: 17–18 %), the dominant class of root GS was formed by aliphatic GS. KIM was separated from the other populations by relatively high concentrations of 4-hydroxyglucobrasicin.

When analyzing all individual GS compounds using RDA (figures not shown), the effect of belowground herbivory on the GS profiles of the root tissues depended on the plant population (RDA, population: *F* = 29.6, *P* = 0.001, 45.6 % explained variation; treatment: *F* = 2.3, *P* = 0.058, 1.6 % explained variation; interaction: *F* = 1.6, *P* = 0.045; 4.5 % explained variation). To better interpret the results, we did pairwise analyses of control and induced root tissues for each of the five populations.

We found there was a significant difference between the GS profiles of control and induced root tissues for DD (RDA: *F* = 2.8, *P* = 0.028, 9.4 % explained variation), KIM (RDA: *F* = 2.8, *P* = 0.033, 9.5 % explained variation), and WIN (RDA: *F* = 3.2, *P* = 0.015, 10.5 % explained variation), but not for SAH (RDA: *F* = 1.7, *P* = 0.13) and OH (RDA: *F* = 0.3, *P* = 0.95). The compounds that contributed most to the separation between control and root fly-induced DD samples were sinigrin, 4-methoxyglucobrassicin, and neoglucobrassicin. Concentrations of the aliphatic GS sinigrin were higher in induced than in control root tissues (*r* = 0.77), whereas concentrations of the indole GS 4-methoxyglucobrassicin and neoglucobrassicin were higher in control than in induced root tissues (*r* = 0.39 and *r* = 0.35, respectively) (Supplemental Fig. A1A). Most GS compounds were higher in induced than in control root tissues of KIM plants, but especially concentrations of 4-hydroxyglucobrassicin (*r* = 0.76), gluconasturtiin (*r* = 0.46), gluconapin (*r* = 0.4), and sinigrin (*r* = 0.39) (Supplemental Fig. A1B). In root tissues of the WIN population, concentrations of the aliphatic GS sinigrin and glucoerucin were high in induced (*r* = 0.94 and *r* = 0.51, respectively) and low in control tissues, but the opposite pattern was noted for the indole GS neoglucobrassicin (*r* = 0.3) (Supplemental Fig. A1C).

### Amino Acids

Nine different amino acids were detected (Table 1) in root tissues of all five populations. Isoleucine was the most dominant amino acid, accounting for more than 80 % of the total amino acid concentrations.

**Fig. 4:**
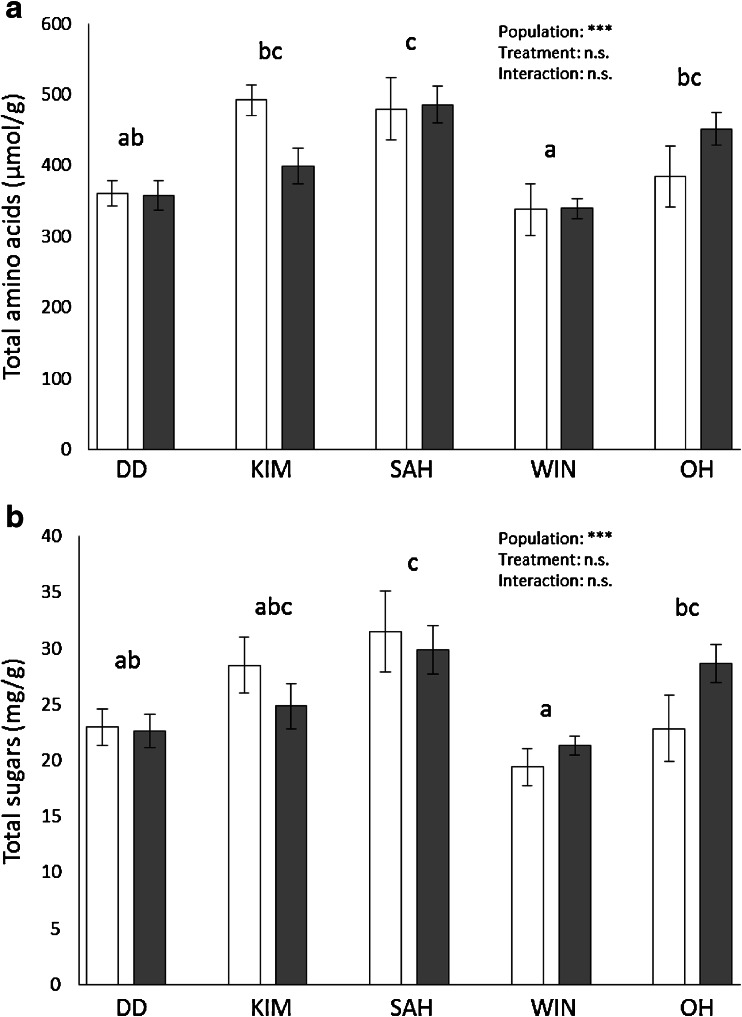
Total concentrations (mean ± SE) of amino acids (**a**) and sugars (**b**) in root tissues of control (*white bars*) and root fly damaged plants (*grey bars*) for the five wild cabbage populations. *** *P* ≤ 0.001, ** *P* ≤ 0.01, * *P* ≤ 0.05, *n.s.* non-significant. *Different letters* indicate significant differences between populations

The total amino acid concentration in roots was significantly affected by plant population (*F*_4,136_ = 8.107, *P* < 0.001), but not by treatment (*F*_1,136_ = 0.016, *P* = 0.90) or by the interaction between plant population and treatment (*F*_4,136_ = 1.248. *P* = 0.29). The lowest amino acid concentrations were found in the root tissues of WIN plants, and the highest concentrations in SAH plants (Fig. 4a).

The amino acid profiles of all wild cabbage populations and both treatments showed no separation based on explorative analysis from the PCA (Fig. 3c, d, but see Supplemental Fig. A2A). However, we did find a separation using RDA: there was a significant effect of plant population (RDA: *F* = 5.7, *P* = 0.001, 13.9 % explained variation) on the amino acid profiles, but not of treatment (RDA: *F* = 1.5, *P* = 0.17) or the interaction between plant population and treatment (partial RDA: *F* = 1.0, *P* = 0.49) (figures not shown). The root tissues of plants from DD had low concentrations of histidine, glutamine, and asparagine, while the root tissues from KIM had high concentration of histidine and also proline and serine. Root tissues from SAH had high concentrations of isoleucine, and tissues from OH had high concentrations of arginine and low concentrations of proline. Root tissues from WIN had low concentrations of all amino acids.

### Sugars

Six different sugars were detected in root tissues of all five plant populations: sucrose, fructose, glucose, sorbitol, mannitol, and trehalose (Table 1). Sucrose was the dominant compound in all populations, making up almost 90 % of the total sugar content.

The total sugar concentration of the roots was significantly affected by plant population (*F*_4_ = 5.932, *P* < 0.001), but not by treatment (*F*_1_ = 0.227, *P* = 0.64) nor by the interaction between plant population and treatment (*F*_4_ = 1.59, *P* = 0.18). Similar to amino acids, the lowest sugar concentrations were found in root tissues of WIN plants and the highest in root tissues of SAH plants (Fig. 4b).

Sugar profiles of all wild cabbage populations and both treatments showed considerable variation based on explorative analysis from the PCA (Fig. 3e, f, but see Supplemental Fig. A2B). Using RDA (figures not shown), we found a significant effect of plant population (RDA: *F* = 2.8, *P* = 0.005, 7.3 % explained variation) on the sugar root profiles. There was no significant effect of treatment (RDA: *F* = 1.8, *P* = 0.016) or interaction between plant population and treatment (RDA: *F* = 1.4, *P* = 0.17). Root tissues of plants from DD had low concentrations of fructose, sorbitol, and sucrose. Root tissues from KIM had low concentrations of glucose and fructose and high concentrations of sucrose. For SAH, root tissues had high concentrations of fructose and sucrose, while root tissues of WIN had low concentrations of sucrose. Root tissues from OH had high concentrations of sorbitol, fructose, and sucrose.

### Root Biomass

Root biomass was similar for all populations (*F*_4_ = 2.098, *P* = 0.084) and both treatments (*F*_1_ = 0.007, *P* = 0.94). The effect of the interaction between population and root fly herbivory also was not significant (*F*_4_ = 1.485, *P* = 0.21).

There was a significant correlation between root biomass and total concentrations of GS, amino acids, and sugars (RDA, *F* = 9.1, *P* = 0.002). Root biomass was positively correlated with amino acids (*r* = 0.29) and sugars (*r* = 0.30), and negatively correlated with GS (*r* = −0.1). There was also a significant correlation between root biomass and individual primary and secondary metabolites (RDA, *F* = 5.6, *P* = 0.001). Root biomass was most positively correlated with the GS sinigrin (*r* = 0.27), the amino acids serine and isoleucine (both: *r* = 0.21), and negatively with the GS neoglucobrassicin (*r* = −0.71) and 4-methoxyglucobrassicin (*r* = −0.37).

### Insect Performance

The survival of *D. radicum* did not differ significantly when reared on plants from any of the five populations (logistic regression: *X*^2^ = 8.75, *d.f.* = 4, *P* = 0.07). Survival was generally low with the highest survival (37 %) on KIM plants (Table 2). Development time and adult body mass of the root flies also did not differ among the plant populations (*F*_4, 39.3_ = 1.40, *P* = 0.25 and *F*_4, 38.6_ = 0.41, *P* = 0.80, respectively), but did differ between the sexes for adult body mass (*F*_1,41.6_ = 5.74, *P* = 0.021), with higher body mass for females.

**Table 2:** Means (±SE) of root fly, *Delia radicum*, survival (%), development time (days) and body mass (mg) for the five wild cabbage populations

Performance parameter	Wild cabbage population				
	DD	KIM	SAH	WIN	OH
Survival	18.33 ± 6.15	36.67 ± 7.98	31.67 ± 7.83	21.67 ± 8.12	18.33 ± 6.15
Development time	34.64 ± 0.81	35.86 ± 0.6	34.74 ± 0.95	32.92 ± 1.03	35.64 ± 0.82
Adult body mass	2.21 ± 0.21	1.93 ± 0.16	2.02 ± 0.16	2.23 ± 0.11	2.13 ± 0.18

We found a significant correlation between the adult body mass of male root flies and amino acids (RDA, *F* = 2.7, *P* = 0.037): the body mass was positively correlated with concentrations of histidine (*r* = 0.68), asparagine (*r* = 0.43), glutamine (*r* = 0.4), and serine (*r* = 0.25). There were no other significant correlations between insect performance parameters and GS, amino acids or sugars.

## Discussion

The results show that primary (amino acids and sugars) and secondary chemistry (GS) in root tissues differed significantly among the five wild cabbage populations that were compared. This supports our first hypothesis that states that, as has been reported for leaves, there also is variation in root chemistry among wild cabbage populations. For GS, the effect of root fly herbivory differed with plant population. We did not find an interaction between population and root fly treatment for the primary metabolites. Both primary and secondary chemistry can change in response to root fly feeding but these changes are metabolite- and plant population-specific. Interestingly, survival and development of *D. radicum* did not differ, but survival was relatively low (on average, 25 % of the flies survived to adulthood) on all five *B. oleracea* populations. Thus, we found no support for our second hypothesis in which we postulated that the performance of the root flies, like that of aboveground herbivores, would differ between the cabbage populations.

As reported before for leaf tissues (Gols et al. 2008b; van Geem et al. 2013), the effect of root herbivory on total and individual concentrations of GS was population-dependent. In several studies on different species, it has been noted that in belowground root tissues, aliphatic GS are usually induced, whereas in aboveground leaf tissues indole GS often increases in response to herbivory (Textor and Gershenzon 2009). Gols et al. (2008b) and Harvey et al. (2011) studied the GS profiles of the leaf tissue of KIM, OH, and WIN and found that indole GS were induced primarily by feeding damage from specialist herbivores, the small and large cabbage white butterfly *Pieris rapae* and *P. brassicae*. Our results suggest that the type of GS class that is induced in response to herbivory may be plant organ-specific, although the identity of the attacking herbivore and various feeding related traits, (*e.g.*, chewer, phloem-feeder, miner; specialist, generalist) also play a role in the type and strength of plant chemical induction (Bezemer and van Dam 2005; Blossey and Hunt-Joshi 2003).

In a previous study, we showed that variation in GS profiles of three populations (KIM, OH, and WIN) is more apparent in aboveground than in belowground tissues (van Geem et al. 2013). In this study, we did not examine aboveground chemistry, but total GS concentrations in the roots were much higher than in the previous study (van Geem et al. 2013). In both studies, the root GS chemistry of the wild cabbage plants was dominated by aliphatic GS, which contributed 55–80 % to the total GS content. The aromatic GS, gluconasturtiin, which is found only in very low concentrations in leaf tissues of the wild cabbage populations (van Geem et al. 2013), is produced in significant amounts in root tissues (12–25 %).

In a study with two *Barbarea vulgaris* chemotypes that were exposed to feeding by *D. radicum*, root GS profiles differed from each other independent of herbivory, but amino acid and sugar profiles did not differ between the chemotypes (van Leur et al. 2008). In our study, both the primary and secondary chemistry differed among the plant populations. However, there are some major differences among the plants used in both studies. Whereas in our study the wild cabbage plants were collected from different populations that are spatially separated, the *B. vulgaris* chemotypes were obtained from a single population (van Leur et al. 2006). This, together with the fact that wild cabbage plants are perennials that live up to 10 years and *B. vulgaris* plants are biennials, suggests that the (a)biotic selection pressures were less diverse for *B. vulgaris* in the van Leur et al. (2008) study than for the wild cabbage studied here. This may explain why profiles for primary metabolites of the two *B. vulgaris* chemotypes did not differ significantly. However, in contrast with our results, van Leur et al. (2008) did find an effect of belowground herbivory on primary metabolites: for both amino acids and sugars, total levels were lower in induced than in control plants. Hopkins et al. (1999) also found that belowground herbivory by *D. radicum* reduced total sugar concentrations in several cabbage cultivars, but this effect differed for individual sugars among cultivars and genotypes. A potentially mitigating factor was that the number of root fly larvae used for inoculation was lower in our study than in the one by Hopkins et al. (1999). Although we found an induction effect for GS, perhaps the number of root fly larvae was too low to stimulate dramatic changes in primary chemistry.

The performance of root flies was not significantly different across the wild cabbage populations, despite the different primary and secondary chemical profiles of the plants. It may be that *D. radicum*, a specialist herbivore that uses GS as oviposition stimulants (Griffiths et al. 2001), is well adapted to a wide range of GS compounds and concentrations, and thus is not negatively affected by these secondary metabolites. On the other hand, survival to adulthood was uniformly low across all populations, which could mean that plants from all five cabbage populations had sufficiently high levels of GS to negatively affect this herbivore. Although differences in plant quality mediated by primary and secondary metabolites may affect development of *D. radicum*, harsh conditions in the soil environment may generate enough variation to mask any potentially deleterious effects of plant quality on the performance of root flies. Previous work with *D. radicum* (Soler et al. 2007) on a related species, black mustard (*B. nigra*), with four root flies per plant, also reported low survival of flies to adulthood, whereas most studies with specialist aboveground feeding herbivores report high survival of the insects (*e.g.*, Gols et al. 2008a, b, 2009). This suggests that the soil is a more hostile environment than the aboveground environment.

We did not find a difference in root biomass between the populations or between control and root fly damaged plants. This may be due to compensatory growth, a tolerance mechanism in which plants invest extra resources into root growth after root tissue damage (Karban and Baldwin 1997). Another possible explanation is that the number of root fly larvae per plant was not high enough to cause significant damage that would lead to a decreased root biomass. However, Soler et al. (2007) did find an effect on root biomass, despite low survival of the root flies.

In summary, we report that five wild cabbage populations that grow along a linear transect of the English Channel coastline within 20 km of one another exhibit significant qualitative and quantitative differences in the expressions of GS, amino acids, and sugars in root tissues. Given that the populations grow in close proximity, it is interesting to speculate about the factors that have generated and maintain these differences in chemical profiles of the roots and shoots. Plants may live up to 10 years in the wild, and, as we have previously reported, some populations (*e.g.*, KIM, SAH) grow on high cliffs where they are continually exposed to prevailing westerly winds, whereas others (*e.g.*, WIN) grow in more sheltered locations. Although the performance of a specialist root-feeding herbivore did not differ across the populations, we cannot exclude the possibility that the observed variation in chemistry has been shaped by differing selection pressures – including climate, and interactions with shoots and root antagonists, such as herbivores and pathogens. Aboveground studies have shown that the abundance and identity of insects on aboveground plant tissues varies among populations (Newton et al. 2009).

A pilot study with three of the populations (WIN, KIM, OH) conducted in a garden plot at the NIOO showed that root flies primarily attack young plants with resulting differential mortality. We do not know what impact *D. radicum* attack has on wild cabbage plants in their natural habitat in Dorset, but it is likely that the insect also will affect the establishment of young plants. Future studies aim to quantify the rate of attack and density of root flies in the wild cabbage populations in England. Work underway also will show whether communities of belowground microbes and nematodes similarly differ between the five populations.

Few studies have examined chemical profiles of roots of different plant genotypes and linked the chemistry to the performance of a belowground herbivore. This study complements extensive previous work of the aboveground chemistry of wild cabbage, and adds to more comprehensive knowledge about wild cabbage populations and their interactions with associated insects.

## Electronic supplementary material

ESM 1 (DOCX 74 kb)
