# Supplementary material for: Interactions Between a Belowground Herbivore and Primary and Secondary Root Metabolites in Wild Cabbage
Source: J Chem Ecol. 2015 Aug 14;41(8):696–707. doi: 10.1007/s10886-015-0605-7 (PMC4568014; doi:10.1007/s10886-015-0605-7)
Supplement: Supplementary file 1 — (DOCX 74 kb) [file 10886_2015_605_MOESM1_ESM.docx]

APPENDIX

**Fig. A1** PCA ordination plots of DD (A), KIM (B) and WIN (C) showing contributions of the Glucosinolate (GS) to the separation of control (downward triangle) and induced (upward triangle) root tissues. The percentage explained variation is given for each axis between parentheses

**Fig. A2** PCA ordination plot of the amino acids (A) and sugars (B) with the five wild cabbage populations projected as explanatory variables. The percentage explained variation is given for each axis between parentheses. DD: star, KIM: triangle, SAH: square, WIN: circle, OH: diamond

Figure A1

PCA axis 1 (39.1%)

PCA axis 2 (23.7%)

PCA axis 1 (36.4%)

PCA axis 2 (28.8%)

PCA axis 1 (46%)

PCA axis 2 (24.9%)

A

B

C

Figure A2

A

B

PCA axis 1 (36%)

PCA axis 1 (64.8%)

PCA axis 2 (20.2%)

PCA axis 2 (26.2%)
